# Imaging insights in veno-venous and veno-arterial extracorporeal membrane oxygenation (ECMO): CT protocols, underlying pathophysiology, and main complications

**DOI:** 10.1186/s13244-025-02100-8

**Published:** 2025-10-26

**Authors:** Francesco Lauriero, Giuseppe Cicchetti, Alessio Perazzolo, Silvia De Vizio, Daniele Perla, Agostino Meduri, Riccardo Marano, Anna Rita Larici, Luigi Natale

**Affiliations:** 1 Advanced Radiology Center (ARC), Department of Diagnostic Imaging, Oncological Radiotherapy and Hematology, Fondazione Policlinico Universitario Agostino Gemelli IRCCS, Rome, Italy; 2 Department of Radiological and Haematological Sciences—Section of Radiology, Università Cattolica del Sacro Cuore, Rome, Italy

**Keywords:** Extracorporeal membrane oxygenation, Veno-venous ECMO, Veno-arterial ECMO, Multidetector computed tomography, Chest X-ray

## Abstract

**Abstract:**

Extracorporeal membrane oxygenation (ECMO) is a vital life support technique employed in patients experiencing pulmonary or cardiopulmonary failure. This procedure entails the use of a pump to replace heart function and an oxygenator to ensure adequate blood oxygenation. ECMO systems are categorized into two main configurations: veno-venous (VV) and veno-arterial (VA) circuits. VV-ECMO is employed for isolated respiratory failure, while VA-ECMO provides temporary mechanical circulatory support for patients with cardiogenic shock or cardiac arrest. A less common alternative, veno-arterial-venous (VAV) ECMO, may be used in complex cases, reducing left ventricular afterload, leading to an improvement of pulmonary edema. Imaging plays a pivotal role in ECMO management, particularly in confirming proper cannula placement, detecting malposition or migration, and identifying complications such as hemorrhage, thrombosis, vascular injury, or infections. Chest X-ray serves as the first-line imaging modality, while computed tomography (CT) is essential for a more detailed evaluation in cases of suspected complications. Tailored CT protocols, adapted to specific ECMO configurations, contrast injection site, cardiac output, and ECMO flow rate, are essential to account for possible ECMO-induced hemodynamic changes and ensure accurate diagnosis. This review provides a comprehensive guide for radiologists, offering detailed descriptions of ECMO system configurations, cannula positioning, and imaging techniques. It highlights the importance of understanding ECMO-specific challenges and outlines strategies to optimize imaging protocols, including modifications in contrast administration and flow-rate adjustments, ultimately improving diagnostic accuracy and patient outcomes.

**Critical relevance statement:**

Radiologists must be familiar with VV- and VA-ECMO systems, utilize tailored CT protocols, and apply non-invasive imaging modalities to assess cannula positioning and complications, ensuring accurate evaluation and management of critically ill patients relying on these advanced life-support techniques.

**Key Points:**

- ECMO is a life support technique used in patients with pulmonary or cardiopulmonary failure.
- CT protocols should be customized based on the study indication, ECMO configuration, contrast injection site, cardiac output, and ECMO flow rate.
- Non-invasive imaging is crucial for evaluating cannula placement and identifying complications.
- Approximately 50% of ECMO patients develop complications, the most frequent being hemorrhage, thromboembolic disease, renal failure, sepsis, and vascular injury.

**Graphical Abstract:**

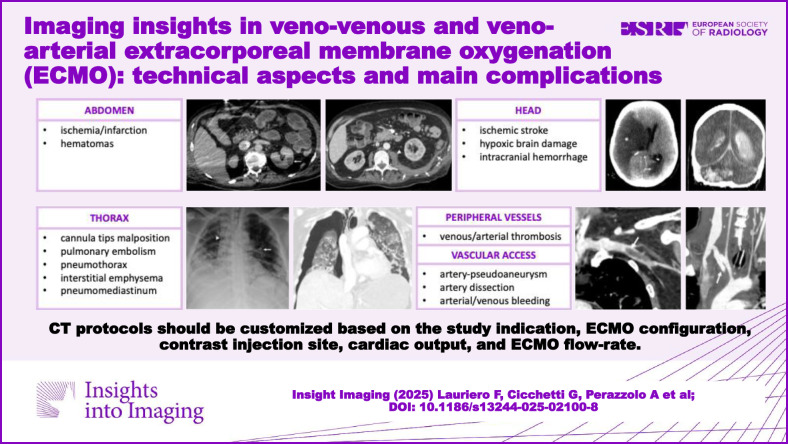

## Introduction

Extracorporeal Membrane Oxygenator (ECMO) is a life support technique used in patients with pulmonary or cardiopulmonary failure, which employs a pump to replace heart function, while an oxygenator is used to provide blood oxygenation, acting as a substitute for the respiratory system [1]. Blood is drained from the vascular torrent through an inflow cannula, then directed outside the body by a mechanical pump, oxygenated via a membrane oxygenator, warmed in a heat exchanger, and ultimately reinfused into the circulation through an outflow cannula [2, 3].

Two main ECMO configurations exist, distinguished by the placement and function of the return cannula: the veno-venous (VV) and the veno-arterial (VA) circuits. VV-ECMO is used for isolated respiratory failure, in which the lungs are unable to ventilate and oxygenate blood despite the use of optimal mechanical ventilation methods and treatment; in this setting, the blood is simultaneously removed and reintroduced into the venous circulation. On the contrary, VA-ECMO is a temporary mechanical circulatory support for patients with cardiogenic shock or cardiac arrest, with or without associated respiratory failure; it bypasses both the heart and the lungs, providing complete hemodynamic support and concomitant gas exchange [4].

Veno-arterial-venous (VAV) ECMO is an alternative modality for selected patients, with limited supporting evidence, usually applied in experienced centers for particular scenarios, such as severe left ventricular failure combined with lung failure. It supplies oxygenated blood to the vital organs in the upper body, especially the brain and myocardium. Moreover, VAV-ECMO allows to reduce the flow in the arterial cannula, resulting in decreased left ventricle (LV) afterload and left atrial pressure, leading to an improvement in the pathophysiologic mechanisms of pulmonary edema [5].

Data gathered from the international registry of the Extracorporeal Life Support Organization (ELSO) indicate that during the biennium 2020–2021 a marked increase in the number of patients receiving ECMO support was registered due to the COVID-19 pandemic [6], relying on the previous successes in critically ill patients diagnosed with other infectious interstitial pneumonitis, such as H1N1 influenza or Middle Eastern respiratory syndrome coronavirus (MERS-CoV) [7, 8]. The great majority of COVID-19 patients requiring ECMO have been supported using VV-ECMO [6, 9]. The use of VA-ECMO has usually been limited to selected COVID-19 patients with cardiogenic shock or indirect cardiovascular complications, including acute myocardial injury, myocarditis, arrhythmias, pericardial effusions, and venous thromboembolism [10]. As expected, the impact of COVID-19 on ECMO application has currently diminished; by 2022, the ELSO Registry showed a decrease in ECMO support, almost returning to pre-pandemic levels of employment [6].

## ECMO types

**Fig. 1:**
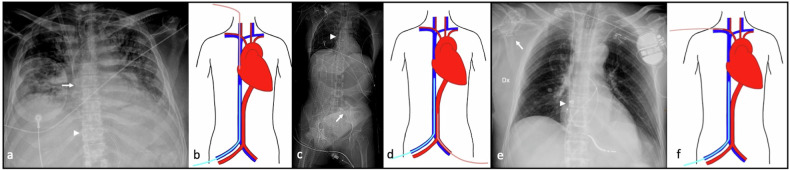
Different extracorporeal membrane oxygenation (ECMO) configuration. **a**, **b** Veno-venous (VV)-ECMO with femoro-atrial configuration: Chest X-ray (CXR) in a COVID-19 patient with bilateral interstitial pneumonia showing the expected locations of the return cannula and the drainage cannula in VV-ECMO with classical femoro-atrial configuration. Cannula tips are located in the right atrium (RA) (arrow) and the RA-inferior vena cava (IVC) junction (arrowhead), approximately 9 cm apart, as illustrated. **c**, **d** Peripheral femoro-femoral veno-arterial (VA)-ECMO: Thoracic-abdominal computed tomography scout-view demonstrates common peripheral VA-ECMO placement, with the venous cannula tip at the superior vena cava-atrial junction and the arterial cannula in the abdominal aorta. **e**, **f** Peripheral subclavian-femoral VA-ECMO: CXR of VA-ECMO with axillary artery access (**e**) shows the arterial cannula in the right axillary artery (arrow) and venous cannula in the RA (arrowhead), as depicted (**f**)

In the usual VV-ECMO configuration (termed femoro-atrial cannulation), the cannula that removes the blood from the circulation (drainage cannula) is introduced through the femoral vein and advanced to the level of the diaphragm, at the inferior vena cava (IVC)-atrial junction. The cannula used for reinfusion (return or reinfusion cannula) is introduced through the internal jugular vein (IJV), with its tip advanced at the superior vena cava (SVC)-atrial junction level, directed toward the tricuspid valve (TV) [11, 12] (Fig. 1). The femoro-atrial cannulation is associated with less recirculation and improved flow, compared to the reverse (atrio-femoral cannulation) configuration [13].

Recirculation is a phenomenon that decreases the VV-ECMO effectiveness, since the oxygenated blood, re-injected by the reinfusion cannula, is not introduced into the systemic circulation, but it is directly withdrawn from the draining venous cannula. The recirculation fraction (*R*_f_) is defined as the portion of oxygenated blood immediately withdrawn by the ECMO circuit without contributing to patient oxygenation [14]. Although there is no standardized threshold, recirculation exceeding 20–30% is typically considered clinically significant [15, 16]. It is important to note that the proximity of the reinfusion and drainage tips directly affects the amount of recirculation, with a higher percentage of recirculated blood flow when the two tips are in closer proximity. To prevent or reduce recirculation, the tip of the drainage cannula should be kept away from the inflow into the right ventricle, and the tips of both cannulas should be adequately apart [14, 17]. Particularly, recirculation is rarely a problem if the tips are ≥ 8 cm distant indeed; however, it should be noted that this cut-off value is empirically derived and not based on randomized clinical trials [18, 19].

In case of challenging IJV cannulation scenarios (e.g., anatomical variants or hypoplasia of IJV), an alternative femoro-femoral configuration for VV-ECMO is being considered. In this configuration, both the drainage and the return cannulas are introduced through the common femoral veins. The drainage cannula is advanced to the IVC, near the hepatic veins, while the tip of the reinfusion cannula is placed in the right atrium (RA) [20]. However, the return blood in this configuration is directed toward the SVC rather than the TV, potentially creating an abnormal flow away from the valve, leading to possible increased recirculation [19].

A less common VV-ECMO configuration is the single-site dual lumen ECMO, characterized by a single cannula inserted through a central venous access site, usually the right internal jugular vein. In this setting, the cannula drains the blood from both the SVC and the IVC and supplies the oxygenated blood to the mid-RA through a slight discontinuity on the cannula, which appears as a radiolucency on chest X-ray (CXR).

On the other hand, the VA-ECMO can be performed through either a peripheral or a central access [21].

The central VA-ECMO is usually considered a separate entity; it requires an open thoracotomy approach and is generally performed in a peri-/postoperative setting after cardiothoracic surgery. It is reserved for patients in acute cardiogenic shock who are unable to be weaned off cardiopulmonary bypass [22, 23]. Typically, the drainage cannula is positioned directly into the right atrium, while the reinfusion cannula is placed into the ascending aorta; in this setup, the reinfused oxygenated blood circulates in a forward, physiologically appropriate direction.

Conversely, peripheral VA-ECMO is principally indicated in case of refractory cardiogenic shock and cardiac arrest, accessing the femoral artery and femoral or internal jugular vein [11]. A notable advantage of peripheral VA-ECMO is its quicker initiation and reduced technical complexity, as it can be started outside of the operating room and even during ongoing chest compressions [24]; its use is rapidly expanding in the context of extracorporeal cardiopulmonary resuscitation (eCPR), where VA-ECMO is initiated during ongoing resuscitative efforts in patients with cardiac arrest [25]. In peripheral VA-ECMO, the tip of the venous drainage cannula is commonly positioned in the mid-RA or at the SVC-atrial junction, or occasionally in the IVC or SVC [26]. Oxygenated blood, subsequent to its passage through the extracorporeal membrane oxygenator, is reintroduced into the systemic circulation via the arterial cannula, typically introduced through the common femoral artery, with its tip located in any position between the external iliac artery and the abdominal aorta (Fig. 1). Currently, no high-level scientific evidence supports one specific location as optimal for the tip of the arterial cannula; its position is usually determined by individual anatomical considerations and technical feasibility, including the potential use of a subclavian approach in selected cases [27].

Compared to central VA-ECMO, this technique is faster and less invasive; however, it creates a non-physiological flow pattern, directing oxygenated blood primarily to the lower body [28]. The blood is infused into the iliac artery and flow is retrograde; therefore, fully saturated blood from the ECMO circuit comes into contact and mixes with blood ejected from the LV in the aorta in an anterograde direction. The location of this mixing point depends on the magnitude of the pressure and blood flow generated by the ECMO pump and the degree of LV ejection. In case of severe myocardial dysfunction, the mixing point is in the proximal ascending aorta or aortic root. As myocardial function improves, the mixing point may migrate more distally into the aortic arch. During severe respiratory failure, the typical VA-ECMO flow rate can result in desaturated blood from the LV, caused by impaired lung function, perfusing the aortic arch and coronary arteries, while fully saturated blood provides sufficient oxygenation of the lower body. This phenomenon is known as “dual circulation,” also identified as “Harlequin syndrome” or “North-South syndrome” [5, 29, 30]. To avoid this phenomenon, the arterial cannula might be placed through the subclavian-axillary arteries (Fig. 1) toward the ascending aorta; this method facilitates a more natural forward flow of the oxygenated blood, reduces the risk of limb ischemia, and increases cerebral oxygen saturation levels [31, 32].

## Imaging modalities in ECMO patients

Non-invasive imaging is crucial in assessing the placement of the cannulas and promptly detecting ECMO-associated complications.

**Fig. 2:**
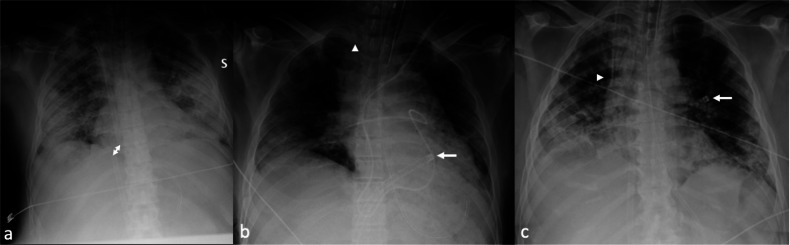
Chest X-ray (CXR) of COVID-19 patients with bilateral pneumonia illustrating veno-venous (VV) extracorporeal membrane oxygenation (ECMO) cannula malposition. **a** Cannula tips are too close, both at the right atrium (RA)-inferior vena cava (IVC) junction (approximately 2.5 cm apart; double-ended arrow). **b** VV-ECMO with mispositioned cannula tips: the drainage cannula tip in the right ventricle (arrow), and the reinjection cannula tip in the right jugular vein (arrowhead). The Swan-Ganz catheter tip is abnormally distal, located in the right interlobar artery. **c** The misplaced drainage cannula shows the tip located in a left superior pulmonary vein (arrow), passing in the left atrium through a patent foramen ovale (PFO), as demonstrated in a subsequent echocardiogram (not shown); the reinjection cannula is correctly positioned at the superior vena cava (SVC)-right atrium junction (arrowhead)

CXR is the first imaging modality to assess the position of the cannula tips, detecting eventual malposition or migration (Fig. 2). Sometimes suspected malposition may require confirmation either by Computed Tomography (CT) or echocardiography. CXR is also pivotal for the initial assessment of complications such as hemothorax, pneumothorax, or—less frequently—mediastinal collections [33].

CT is typically conducted when there is a suspicion of complications such as bleeding or thrombosis. Despite the importance and wide application of CT, a standardized and internationally agreed protocol for ECMO patients’ evaluation is still lacking. Protocols should be tailored to the study indication, ECMO configuration, contrast injection site, cardiac output, and ECMO flow rate [26]. In addition, particular attention must be paid to the potential ECMO-induced hemodynamic changes (Table 1).

**Table 1:** Tips for appropriately tailoring the CT protocols according to the different ECMO configurations

ECMO configuration		Cardiac output	Systemic CTA		Pulmonary CTA	Venous phase
VA-ECMO	• Central access • Peripheral access (upper body)	Any	• Contrast injection through the oxygenator inlet (anterograde filling) • No ECMO flow changes	ROI at the site of the target arterial vessel	• Contrast injection through the central venous line • Reduce ECMO flow as much as possible (reduce pulmonary circulation bypass)	• Contrast injection through the oxygenator inlet • No ECMO flow changes
	Peripheral access (femoral)	Preserved cardiac output	• Contrast injection through the central venous line • Reduce ECMO flow			
		Low cardiac output	• Contrast injection through the oxygenator inlet (retrograde filling) • No ECMO flow changes			
VV-ECMO	Any access and cannula position	Any	• Contrast injection through the oxygenator inlet (anterograde filling); no ECMO flow changes • Contrast injection through central venous line; reduce ECMO flow (decrease recirculation)			

*ECMO* extracorporeal membrane oxygenation, *CT* computed tomography, *CTA* computed tomography angiography, *VA* veno-arterial, *VV* veno-venous, *ROI* region of interest

In VV-ECMO, dynamic CT images are comparable to those of healthy individuals [34]. Nevertheless, the oxygenated blood flows in an antegrade manner into the RA, where it mixes with the centrally injected contrast agent; the simultaneous potential withdrawal of contrast medium by the drainage cannula reduces the diagnostic quality of enhancement in the pulmonary arteries [26, 35]. To mitigate these issues, it is preferable to administer contrast medium through the return cannula within the ECMO machine and increase the contrast medium volume and flow rate or temporarily reduce the ECMO flow rate [36]; additionally, contemplating the complete temporary cessation of VV-ECMO during the CT acquisition period is advisable [26, 33].

On the contrary, in VA-ECMO, the enhancement of the vessels of both systemic and pulmonary systems depends on the preserved cardiac pump function [26, 35, 37, 38]. Given the profound hemodynamic instability and highly dynamic condition of these patients, cardiac output may fluctuate significantly; therefore, the most up-to-date cardiac output measurement should always be considered to avoid unexpected outcomes when tailoring CT protocols based on anticipated flow dynamics is performed.

In case of a partially sustained cardiac pump, the contrast medium is injected in a central vein, then passes through the pulmonary circulation and flows in an antegrade manner into the aorta. This results in mixing the contrast-enhanced antegrade blood with the non-contrast-enhanced extracorporeal oxygenated blood, resulting in a possible arterial pseudo-filling defect. To address these issues, it is advisable to decrease the ECMO flow rate—if deemed safe—during the acquisition phase [36, 39, 40]. Attention should be given to the use of high-pitch acquisition as the pump may be displaced due to rapid table excursion [39].

**Fig. 3:**
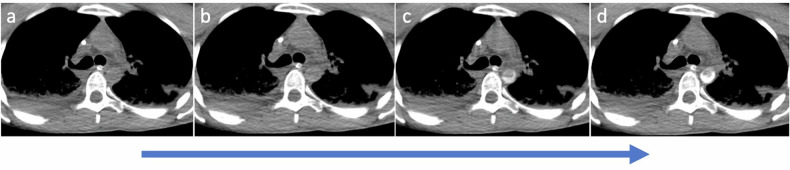
Computed tomography (CT) bolus tracking at the level of the distal ascending aorta in a femoro-femoral veno-arterial (VA)-extracorporeal membrane oxygenation (ECMO) supported patient showing retrograde contrast flow with the enhancement of the descending aorta before the ascending aorta. Contrast-blood layering is visible in the descending aorta (**c**, **d**), whereas no enhancement of the ascending aorta is identified in the earlier phases (**a**, **b**)

**Fig. 4:**
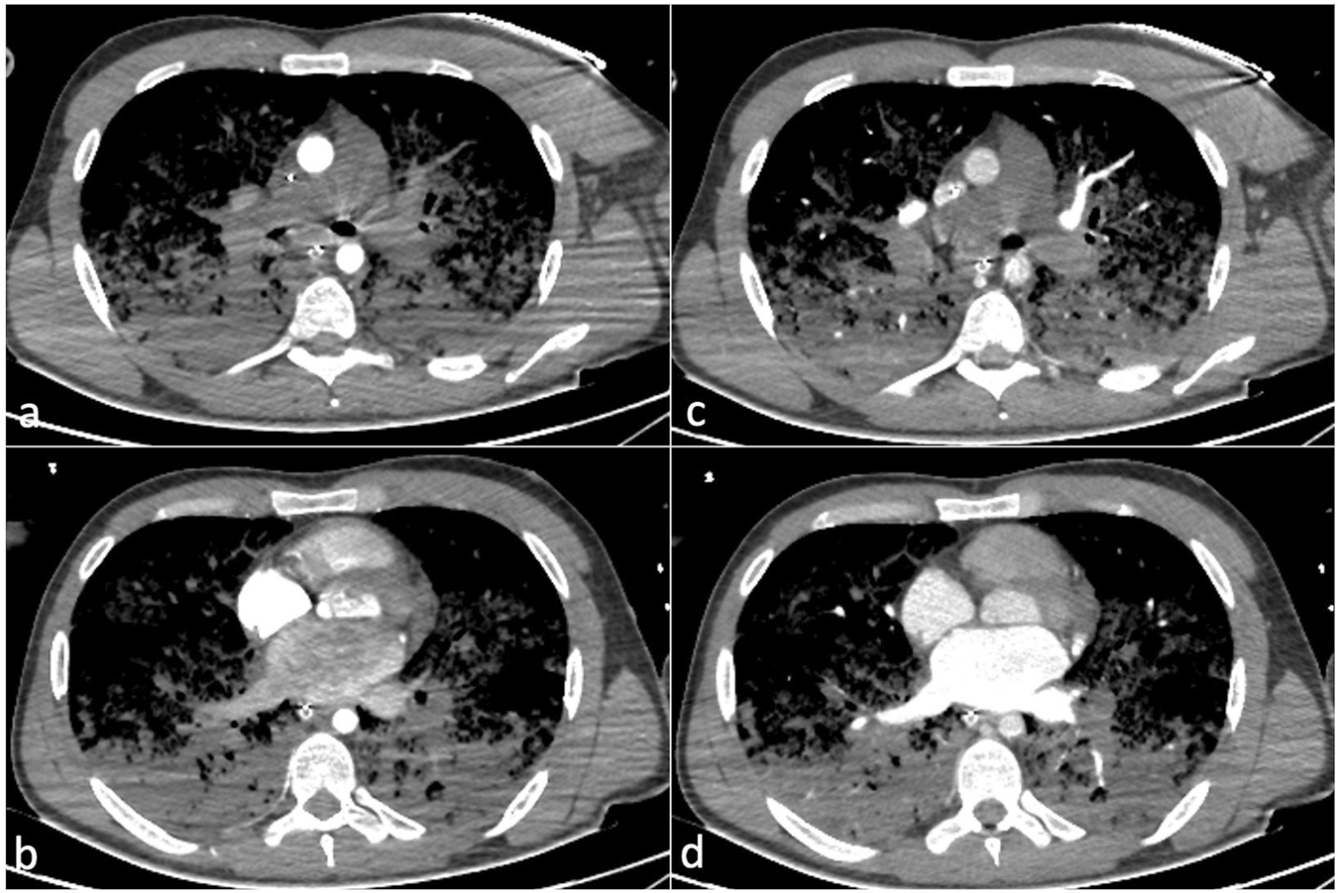
Axial contrast-enhanced computed tomography (CT) images in the arterial (**a**, **b**) and venous phases (**c**, **d**) in an acute respiratory distress syndrome (ARDS) patient on veno-arterial (VA) extracorporeal membrane oxygenation (ECMO) therapy with severe heart failure. At the main pulmonary artery bifurcation (**a**, **c**), there is no enhancement in the pulmonary arteries, while at the left atrium level (**b**, **d**), retrograde filling of the left atrium and pulmonary veins is seen due to the VA-ECMO system

**Fig. 5:**
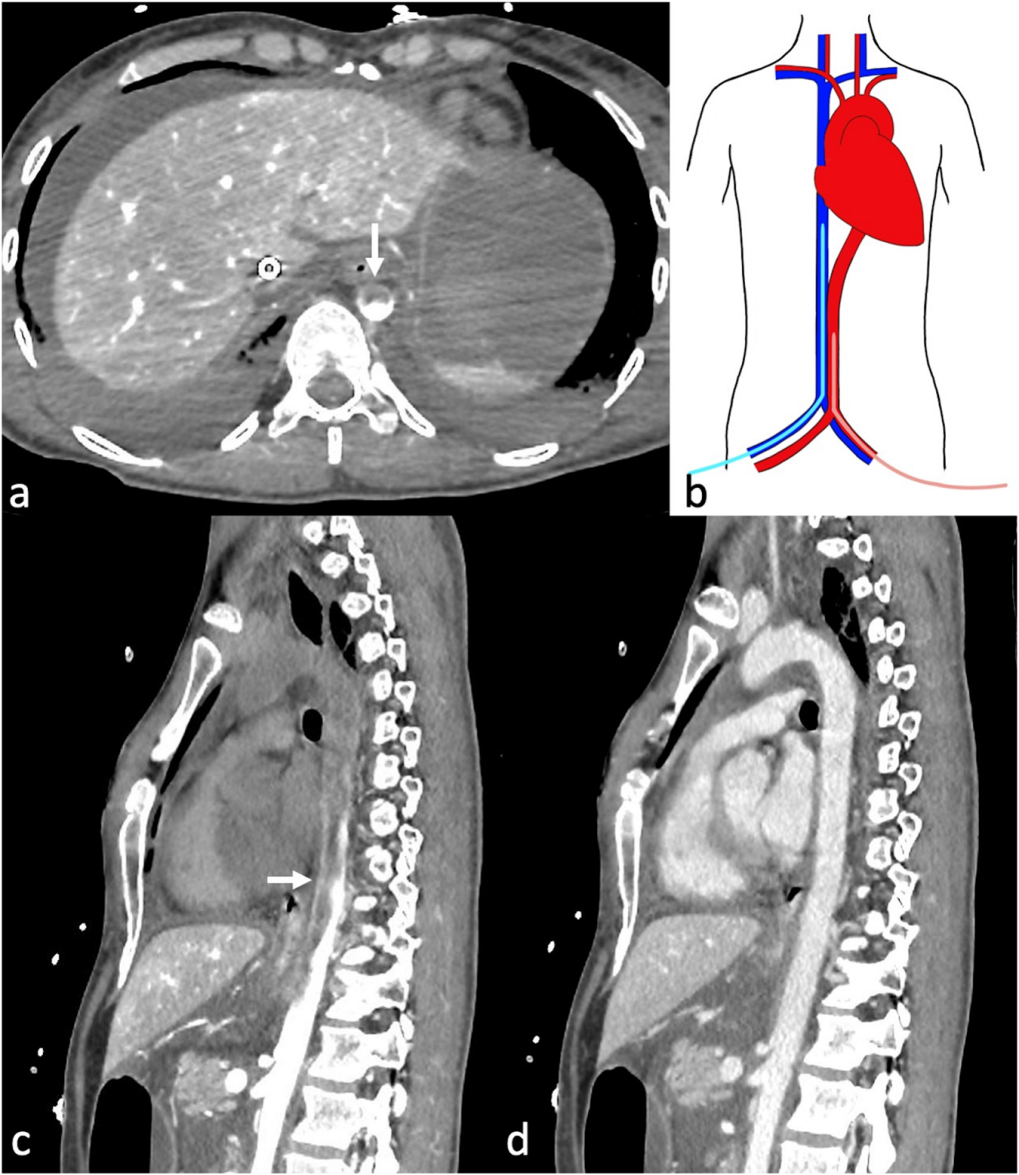
Axial (**a**) and sagittal (**c**) computed tomography (CT) images in the arterial phase of a veno-arterial (VA) extracorporeal membrane oxygenation (ECMO) supported patient with classic femoro-femoral configuration (**b**), showing the “arterial pseudomembrane” phenomenon. This occurs in severe heart failure cases, where enhanced blood from the femoral artery meets the low-enhanced blood from the heart. In such instances, a delayed phase (**d**) is required to prevent the misdiagnosis of an aortic dissection

Otherwise, in case of severely compromised heart function, the contrast medium flows in a retrograde manner through the venous drainage cannula into the extracorporeal circuit, then directly into the arterial system. This is clearly evident during the bolus tracking at the level of the aortic arch, where—in case of typical femoro-femoral VA-ECMO configuration—a dynamic of reversed systemic flow in the aorta can be appreciated (Fig. 3). This results in a lack of complete enhancement of both the right heart and the pulmonary vascular system (Fig. 4). In case of severely compromised heart function, if the CT scan is acquired before complete enhancement of the left heart chambers, the discrepancy in densities between non-contrast-enhanced and contrast-enhanced blood may lead to a sedimentation phenomenon in the aorta. This can mimic a dissection flap or intramural hematoma (phenomena referred to as “watershed” or “arterial pseudomembrane”). These occur at the interface where retrograde ECMO flow meets antegrade flow from the left ventricle, with the watershed level determined by the relative pressures and flow rates of the two systems [39]. However, in the venous (“late”) phase, this phenomenon is mitigated due to contrast recirculation and homogenization, which is why a venous or delayed phase is generally recommended in patients supported with VA-ECMO to reduce the risk of misinterpretation related to flow-dependent enhancement alterations [26, 39] (Fig. 5).

To obtain a high-quality systemic arteries enhancement during ECMO, particularly when targeting specific vascular territories, Shen et al recommended employing automated bolus tracking with the region of interest (ROI) placed at the site of the target arterial vessel, along with an increased volume of administered contrast [39]. However, despite these precautions, hemodynamic alterations associated with ECMO may still impair optimal opacification of the target vessels during the early angiographic phase. In such scenarios, acquiring an additional delayed or venous phase becomes essential to assess vessel opacification after contrast recirculation and homogenization. This consideration is particularly relevant for cerebral arterial territories, where asymmetrical enhancement between hemispheres is frequently observed in both subclavian-femoral and femoro-femoral peripheral VA-ECMO configurations. Typically, in patients with severely compromised cardiac function, the left cerebral hemisphere demonstrates better opacification during the early arterial phase in femoro-femoral VA-ECMO, likely due to preferential filling of the left common carotid artery via retrograde aortic flow. Conversely, in subclavian-femoral configurations, the hemisphere homolateral to the arterial cannula (usually the right) shows greater enhancement, owing to its direct proximity to the arterial inflow from the ECMO circuit. In this context, late or venous phase brain imaging is often necessary to ensure complete and symmetrical enhancement of both hemispheres, which may otherwise appear uneven or incompletely opacified during the arterial phase [39].

Conversely, for the pulmonary arterial vessels, it is advisable to decrease the ECMO flow rate as much as possible [40]; leaving a minimal flow in the ECMO system allows to prevent thrombus formation in the cannula [33]. However, if the desired pulmonary artery opacification is not achieved with this method, a complete switch-off of the ECMO for the duration of CT acquisition should be considered. Furthermore, a supplementary late phase with weaker but uniform contrast enhancement or a diagnostic angiography can confirm the artifactual nature of contrast enhancement defects. In cases where the only parenchymal representation is sufficient, such as when only a venous phase is required, the contrast medium should be injected through the oxygenator, thus allowing for the maintenance of ECMO flow across all cannulation strategies [36, 41].

To date, the application of magnetic resonance imaging (MRI) in this patient category is not allowed, due to incompatibility with the ECMO equipment [42]. Nonetheless, MRI studies on animals have been performed, indicating possible MRI compatibility and MRI safety [43].

US plays a pivotal role in the planning and execution of ECMO cannulation procedures. It allows for accurate identification of vascular structures, delineation of anatomical relationships, and precise measurement of peripheral vessel diameters, which are for selecting the appropriate cannula size. During percutaneous ECMO cannulation, real-time US guidance facilitates accurate puncture of the target vessel, thereby minimizing the risk of local complications [44]. Moreover, US is instrumental in the early detection of peri-procedural complications, including hematomas, arterial or venous occlusion, and impaired perfusion of distal branches adjacent to the access site [33, 45].

Echocardiography (trans-esophageal and also trans-thoracic) is crucial for patient selection, guiding cannula insertion and positioning, monitoring progression, and detecting complications [46].

Echocardiography has an important role in providing data assisting patient’s selection, guiding the insertion and the exact positioning of the cannula tip, as well as monitoring progression and detecting eventual local complications, including cannula malpositioning [46, 47]. Furthermore, the utilization of trans-esophageal echocardiography as a guide for ECMO cannulation is strongly advised, even though trans-thoracic echocardiography at times provides adequate visualization of cardiac structures [20].

## Complications

Nearly half of ECMO patients experience complications, the most prevalent being hemorrhage, thromboembolic disease, renal failure, sepsis, and vascular injury [48].

The interconnected extracorporeal circuit leads to thrombocyte activation, induces coagulation factor consumption, and triggers a diffuse inflammatory response [49]. This process can ultimately lead to the development of disseminated intravascular coagulation (DIC). In the context of ECMO, a balance of systemic anticoagulation is the basis to prevent either thromboembolic complications or bleeding.

**Fig. 6:**
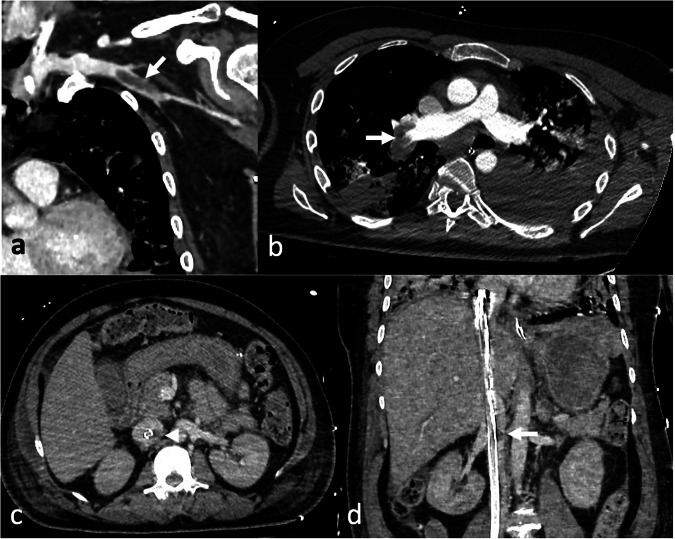
Coronal computed tomography (CT) image in the venous phase (**a**) depicting a thrombotic filling defect in the left axillary vein (arrow), in a veno-venous (VV) extracorporeal membrane oxygenation (ECMO) supported patient. Axial pulmonary CT angiography in a 51-year-old male with acute respiratory distress syndrome (ARDS) (**b**), performed after ECMO cannula removal due to malpositioning, reveals an intraluminal filling defect in the right pulmonary artery and right interlobar artery (arrow), indicating central pulmonary embolism. Venous phase axial (**c**) and coronal (**d**) CT images in a 34-year-old male with femoro-atrial VV-ECMO demonstrate a focal thrombus attached to the drainage cannula at the left renal vein-inferior vena cava (IVC) junction (arrowhead in **c**, arrow in **d**)

With the aid of the aforementioned technical precautions, pulmonary and systemic CT angiography followed by a venous phase are usually performed to exclude thrombotic or thromboembolic complications (Fig. 6). Recently, thromboembolic complications have decreased due to the use of biocompatible materials in the ECMO systems; nevertheless, the incidence of deep vein thrombosis is estimated at 53%, and it is more prevalent in VV-ECMO than in VA-ECMO [50].

**Fig. 7:**
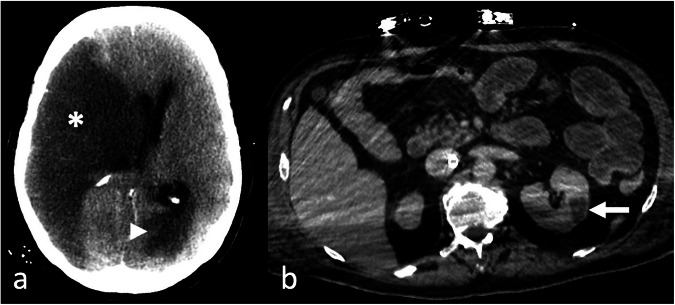
Unenhanced head computed tomography (CT) (**a**) in a 55-year-old patient on extracorporeal membrane oxygenation (ECMO) therapy, shows an extensive ischemic area in the right middle cerebral artery (MCA) territory (asterisk) and a smaller ischemic area located in the left parieto-occipital region (arrowhead), territory of the left posterior cerebral artery (PCA); increased attenuation represents a pseudo-subarachnoid hemorrhage, secondary to engorgement of venous structures due to increased intracranial pressure. Axial CT image in the venous phase (**b**) of a 65-year-old male with femoro-atrial veno-venous (VV)-ECMO reveals a hypoattenuating area in the left kidney (arrow), indicating ischemic damage

Due to the blood-surface interaction, blood clots can form within the circuit, leading to embolic events [51]. Therefore, ischemic issues are frequent complications, caused by either acute arterial thrombosis, embolization, or critical hypoperfusion (Fig. 7). In particular, ischemic stroke is a relatively frequent complication. Unenhanced CT of the brain is the primary imaging modality in this setting, and its sensitivity in detecting ischemic areas depends on the affected territory, radiologists’ expertise, and time elapsed since the onset of symptoms. On the other hand, CT angiography is the preferred modality for detecting intracranial arterial filling defects [52]. Due to diminished cardiac ejection fraction or acidosis associated with therapy, the occurrence of diffuse hypoxic brain damage is also possible [53].

**Fig. 8:**
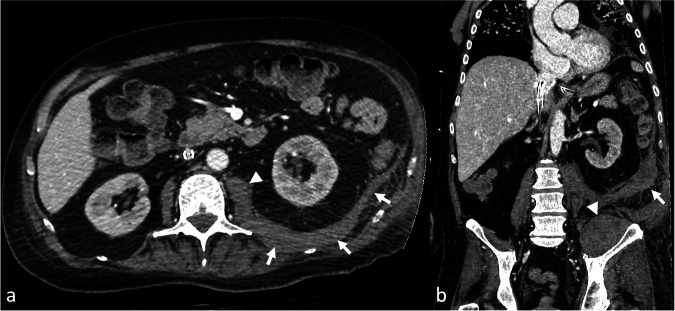
Axial (**a**) and coronal (**b**) computed tomography (CT) images in the venous phase of a 66-year-old male veno-venous (VV) extracorporeal membrane oxygenation (ECMO) supported patient show an extensive left retroperitoneal hematoma (arrows) extending to the ipsilateral psoas muscle (arrowhead). A subsequent angiography did not identify any active bleeding source, as the hemorrhage had spontaneously resolved

**Fig. 9:**
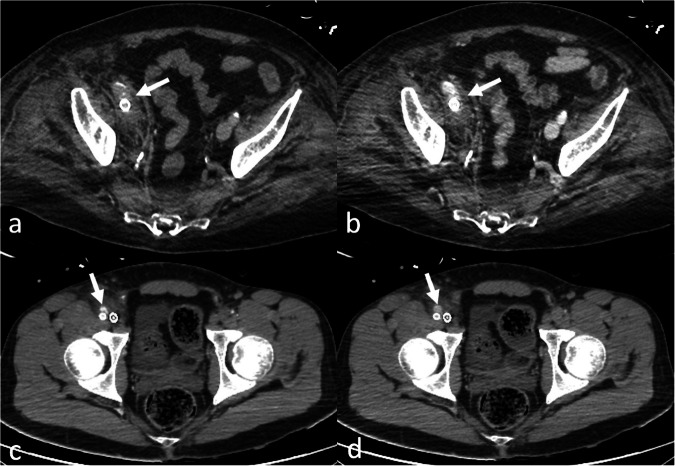
Axial computed tomography (CT) images unenhanced (**a**) and in the venous phase (**b**) reveal a blood collection adjacent to the right iliac vein (arrows), near the drainage cannula. In **b**, there are no signs of active contrast medium extravasation. Axial CT images in the arterial (**c**) and late (**d**) phases show a pseudoaneurysm of the right common femoral artery at the site of arterial return cannula access for veno-arterial (VA) extracorporeal membrane oxygenation (ECMO) (arrows), surrounded by a perivascular hematoma

Bleeding can occur everywhere in the body, most frequently caused by iatrogenic injuries. For this reason, special attention should be paid to the cannulation sites, not only for bleeding detection but also for identifying possible perivascular blood collection (hematomas) or artery pseudoaneurysms (Figs. 8, 9), as arterial cannulation may lead to these complications, especially when a percutaneous technique is used, regardless of the use of US-guided strategy [54–56].

**Fig. 10:**
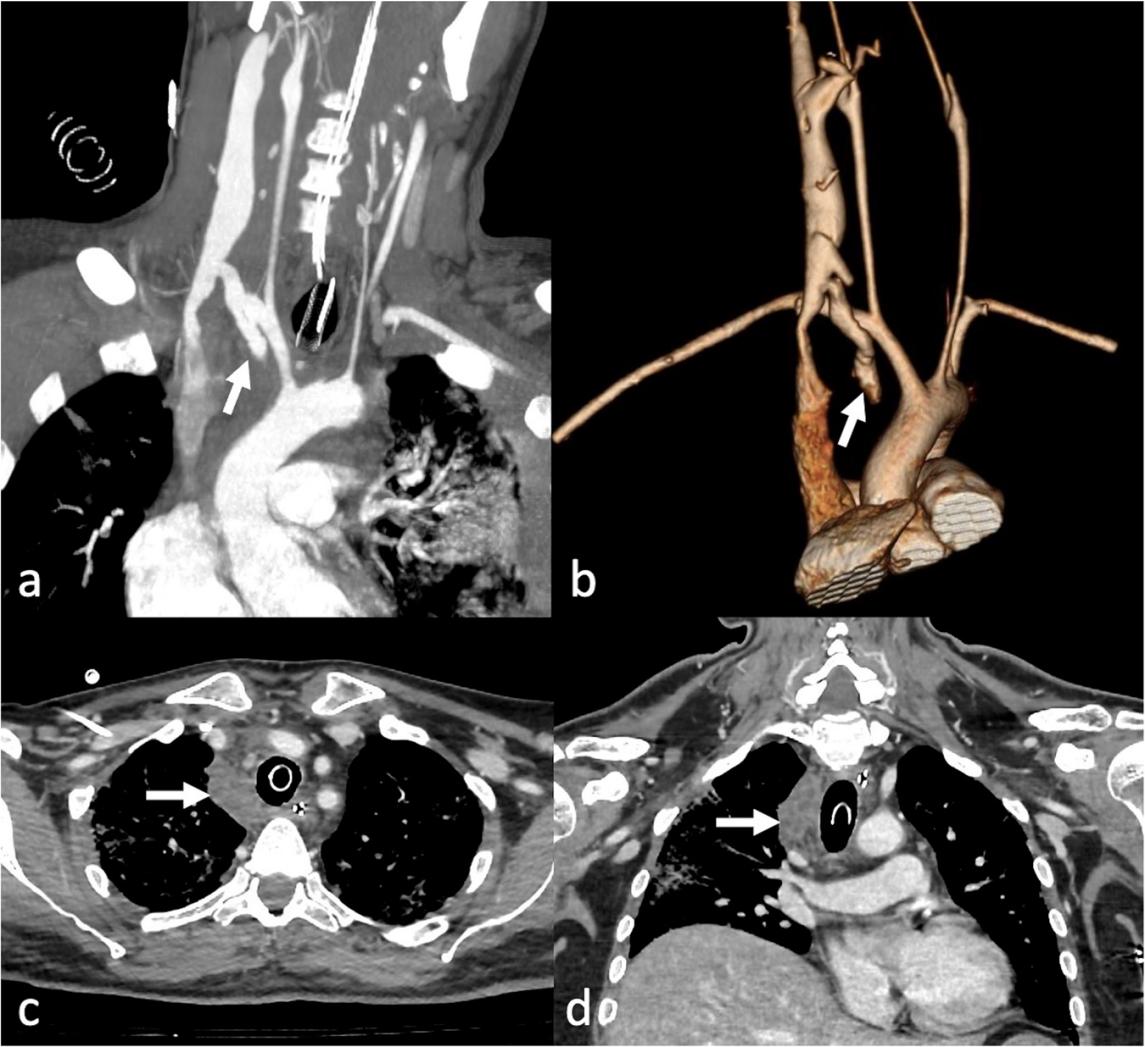
Coronal computed tomography (CT) images in the venous phase, with maximum intensity projection (MIP) reconstruction (**a**) and 3D volume rendering (3D-VR) reconstruction (**b**), show contrast medium extravasation (arrows) due to incorrect cannulation of the right jugular vein, causing vessel wall rupture. Axial (**c**) and coronal (**d**) CT images in the venous phase of the same patient demonstrate a blood collection in the right paratracheal space (arrow)

Active bleeding can be both arterial and venous in origin, and on CT imaging, it is characterized by spreading of contrast medium outside the vessel lumen, with arterial bleeding usually visible in the early contrast-enhanced phase (Fig. 10).

**Fig. 11:**
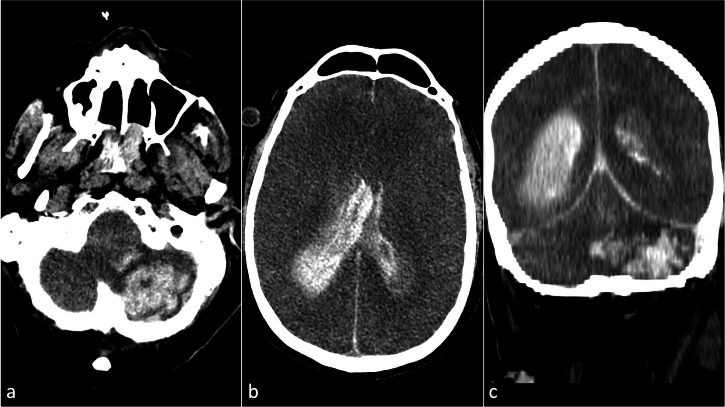
Unenhanced axial (**a**, **b**) and coronal images (**c**) computed tomography (CT) images in an extracorporeal membrane oxygenation (ECMO) supported patient show extensive spontaneous hemorrhage in the left cerebellar hemisphere (**a**, **c**), surrounded by vasogenic edema. Additionally, intraventricular hemorrhage is present in both lateral ventricles, along with a subdural hematoma along the falx cerebri and the tentorium cerebelli (**b**, **c**)

Another frequent complication is spontaneous intracranial hemorrhage (ICH), which is associated with a poor prognosis and is often fatal. ICH is easily detectable on unenhanced CT as an intra-axial collection, hyperdense compared to the brain tissue and the cerebrospinal fluid, often surrounded by a hypodense area of vasogenic edema (Fig. 11) [57, 58]. Pre-ECMO factors can correlate with the incidence of ICH, especially the occurrence of a cardiac arrest before the mechanical support, which may be linked with the development of ICH due to anoxic brain injury or brain infarction with subsequent hemorrhagic infarction [59–61]. Other factors influencing ICH incidence are sepsis, renal failure, hemolysis, thrombocytopenia, younger age, female gender, and lower body surface area [62, 63]. A critical consideration when interpreting hemorrhage or suspected bleeding on CT, especially non-contrast CT of the brain, is the potential presence of residual contrast from recent iodinated contrast-enhanced procedures, such as cardiac catheterization, coronary angiography, or cerebral angiography. This persistent contrast may mimic or obscure true pathology, leading to potential diagnostic pitfalls. When available, Dual-Energy CT (DECT) or MRI can help distinguish between residual contrast and true hemorrhage, although these modalities are not always accessible in emergency settings [64, 65].

The large caliber of the cannulas inserted in placed peripheral vessels poses a significant risk of another major complication in patients undergoing VA-ECMO, represented by limb ischemia. Limb ischemia has a multifactorial genesis, and it can occur at any stage of the ECMO procedure, such as upon cannulation, during support, and upon or following decannulation. The principal mechanism is the reduced blood flow and consequent oxygen supply, which may result from multiple factors, as arterial cannula occlusion, selective perfusion of the deep femoral artery, vessel damage during cannulation, inadequate peripheral perfusion, high vasopressor levels, extrinsic compression by the cannulas, or even atherosclerosis (particularly in the absence of collateral circulation) [66–68]. To prevent this complication, the preferred strategy is the use of a distal perfusion cannula, usually placed within the proximal superficial femoral artery [69].

Infections are common complications in patients treated with ECMO systems, due to several predisposing factors, such as multiple comorbidities, immunocompromised status, and critical illness, among others [51]. Patients with prolonged ECMO duration are prone to develop sepsis, particularly following ventilator-associated pneumonia (VAP) and bloodstream infections. Recognizing predisposing factors and establishing the best preventive strategies and therapeutic choices are critical to optimizing the management of these complications [70]. In this context, nosocomial infections should be primarily identified, often aided by laboratory data, such as white blood cell (WBC) counts, and inflammation indices like C-reactive protein (CRP) and procalcitonin. Furthermore, imaging findings (i.e., onset of lung parenchymal opacities in CXR, suggestive of pneumonia) can play an important role in assessing the diagnosis.

**Fig. 12:**
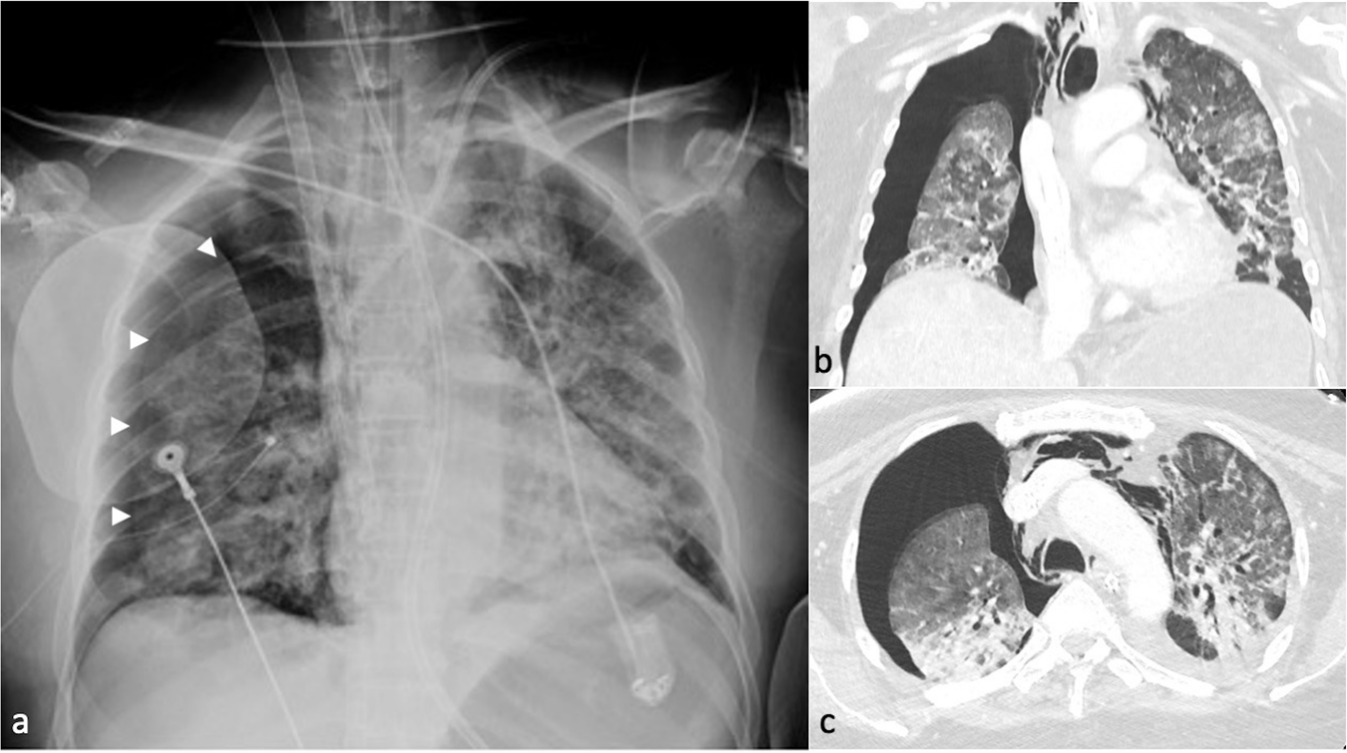
Femoro-atrial veno-venous (VV) extracorporeal membrane oxygenation (ECMO) in a patient with bilateral interstitial pneumonia due to SARS-CoV-2 infection. Chest X-ray (CXR) (**a**) after cannulation shows a significant right pneumothorax with partial lung collapse (arrowhead) and pneumomediastinum, particularly in the upper and mid-third of the mediastinum on the right, near the para-caval area. The distance between the cannula tips is less than 8 cm. Chest computed tomography (CT) in lung window setting (**b**, **c**) confirms the right pneumothorax, causing partial collapse of the right lung, and pneumomediastinum

Pneumothorax can be the consequence of mechanical ventilation or can be accidentally caused during neck vessels cannulation. CXR is usually the first imaging modality, as it can identify pneumothorax—even unexpectedly—when performed to assess the position of the cannula tips (Fig. 12). CXR is capable of detecting a possible associated mediastinal shift in case of tension pneumothorax, which represents an emergency. In addition, mechanical ventilation could also be a cause of barotrauma, with evidence of interstitial emphysema, pneumomediastinum, and eventually, pneumothorax, due to the Macklin effect [71].

Renal failure is another clinically relevant complication in ECMO patients, typically resulting from a multifactorial pathophysiology involving systemic hypoperfusion, hemolysis, sepsis, and exposure to nephrotoxic agents, including contrast media [72]. From an imaging standpoint, decreased or absent nephrographic enhancement on CT may be an indirect indicator of acute kidney injury (AKI), especially when associated with perirenal stranding or delayed contrast excretion [73–75]. Although the use of iodinated contrast media is sometimes restricted due to concerns about contrast-induced nephropathy (CIN), in critically ill patients, including those on ECMO, where hemodynamic instability plays a predominant role in the pathogenesis of AKI, contrast-enhanced CT should not be deferred when clinically indicated [76].

## Conclusion

When dealing with ECMO-supported patients, radiologists should be familiar with the function of the most commonly used VV-ECMO and VA-ECMO systems. Imaging plays a pivotal role in ECMO management, especially in confirming proper cannula placement, detecting malposition or migration, and identifying complications such as hemorrhage, thrombosis, vascular injury, or infections. CXR serves as the first-line imaging modality, while CT is essential for a more detailed evaluation in cases of suspected complications. Tailored CT protocols that account for ECMO-induced hemodynamic changes are essential to ensure accurate diagnosis and detect possible complications in order to improve patient clinical management and outcomes.

## Data Availability

Not applicable. This educational review is based on previously published studies, literature, and clinical experience.

## Abbreviations

CT: Computed tomography
CTA: Computed tomography angiography
CXR: Chest X-ray
ECMO: Extracorporeal membrane oxygenation
ELSO: Extracorporeal Life Support Organization
ICH: Intracranial hemorrhage
IJV: Internal jugular vein
IVC: Inferior vena cava
LV: Left ventricle
MRI: Magnetic resonance imaging
RA: Right atrium
ROI: Region of interest
SVC: Superior vena cava
TV: Tricuspid valve
US: Ultrasound
VA: Veno-arterial
VAV: Veno-arterial-venous
VV: Veno-venous
